# Functions and Molecular Mechanisms of Deltex Family Ubiquitin E3 Ligases in Development and Disease

**DOI:** 10.3389/fcell.2021.706997

**Published:** 2021-08-25

**Authors:** Lidong Wang, Xiaodan Sun, Jingni He, Zhen Liu

**Affiliations:** ^1^Department of General Surgery, Shengjing Hospital of China Medical University, Shenyang, China; ^2^Postdoctoral Research Workstation, Jilin Cancer Hospital, Changchun, China

**Keywords:** Deltex family proteins, ubiquitination, ubiquitin E3 ligase, ubiquitin code, protein homeostasis, post-translational modification

## Abstract

Ubiquitination is a posttranslational modification of proteins that significantly affects protein stability and function. The specificity of substrate recognition is determined by ubiquitin E3 ligase during ubiquitination. Human Deltex (DTX) protein family, which functions as ubiquitin E3 ligases, comprises five members, namely, DTX1, DTX2, DTX3, DTX3L, and DTX4. The characteristics and functional diversity of the DTX family proteins have attracted significant attention over the last decade. DTX proteins have several physiological and pathological roles and are closely associated with cell signal transduction, growth, differentiation, and apoptosis, as well as the occurrence and development of various tumors. Although they have been extensively studied in various species, data on structural features, biological functions, and potential mechanisms of action of the DTX family proteins remain limited. In this review, recent research progress on each member of the DTX family is summarized, providing insights into future research directions and potential strategies in disease diagnosis and therapy.

## Introduction

Intracellular protein homeostasis, i.e., proteostasis, is influenced by the dynamic equilibrium between protein synthesis, localization, maintenance, and degradation, all of which are regulated by protein-protein interaction networks (Zhong et al., 2019). Dysregulated proteostasis is associated with cellular dysfunction and can lead to disease onset, including neurodegeneration (Kaushik and Cuervo, 2015) and cancer (Dai and Sampson, 2016). Ubiquitination is a prominent and highly conserved post-translational modification (PTM) of proteins, during which ubiquitin (Ub) molecules are attached to a target protein. A majority of intracellular proteins are modified by ubiquitination (Amm et al., 2014). Several Ub signals are recognized by proteasomes, thereby serving as a regulatory mechanism for protein degradation, affecting nearly all aspects of cellular processes (Chowdhury and Enenkel, 2015; Hanna et al., 2019; Sakai et al., 2020; Fhu and Ali, 2021; Goetzke et al., 2021; Qu et al., 2021; Zou and Lin, 2021). Ubiquitin signaling is strictly regulated by a multistep cascade reaction consisting of three enzyme groups. Initially, energy from adenosine triphosphate (ATP) hydrolysis is used by the ubiquitin-activating enzyme (E1) to generate a high energy thioester bond between the C-terminus of Ub and a catalytic cysteine residue of the active site in E1. Next, Ub is transferred from E1 to a cysteine residue in the active site of the ubiquitin-conjugating enzyme (E2), forming a similar thioester bond to that of E1. Finally, ubiquitin ligase (E3) catalyzes the covalent attachment of Ub to lysine residues of the substrate protein (Thapa et al., 2020). E2 and/or E3 enzymes are also associated with the elongation of Ub chains (Dikic et al., 2009). Ub contains 76-amino acids with seven lysine residues (Lys6, 11, 27, 29, 33, 48, and 63) and a methionine residue (Met1), all of which can be ubiquitinated and attached to numerous linkage types of Ub chains via an isopeptide bond (Ikeda and Dikic, 2008). Ub ends with a diglycine motif, which is critical for attachment to substrate proteins (Hanna et al., 2019). Monoubiquitination is the attachment of a single Ub molecule to a single Lys of the target protein, which regulates several aspects of protein function, including subcellular localization and protein-protein interaction, in both normal and disease states (Sewduth et al., 2020). Conversely, polyubiquitin (polyUb) chains can be formed on a single Lys by attachment of multiple Ub molecules through internal Ub–Ub linkages (Akutsu et al., 2016); hence, the different types of polyUb chains depend on the Lys for the Ub linkage (Komander and Rape, 2012). In homotypic polyUb chains, a total of eight different chain types can be formed; meanwhile, heterotypic polyUb chains comprise mixed and branched types, containing two or more linkages (Pickart and Fushman, 2004; Kliza and Husnjak, 2020). Among these polyUb chains, Lys48-linked polyUb chains are primarily involved in protein degradation by proteasomes, whereas Lys63-linked polyUb chains are more associated with non-degradative processes, such as vesicular trafficking (Trempe, 2011; Matyskiela and Martin, 2013). Lys63-linked polyUb chain also influences the induction of autophagy, a lysosome mediated protein degradation process (Chen et al., 2019). The linear homotypic polyUb chains are Met1-linked and assembled by a multi-subunit complex referred to as linear Ub chain assembly complex (LUBAC) (Kirisako et al., 2006). Several signaling cascades, such as tumor necrosis factor (TNF) and nuclear factor kappa-light-chain-enhancer of activated B cells (NF-κB), which are involved in immune and inflammatory diseases, are regulated by linear Ub chains (Rittinger and Ikeda, 2017).

More than 600 E3 ligases have been identified in humans. Based on their characteristic catalytic domains and the mechanisms underlying Ub transfer to target proteins, E3s are divided into three major types, namely, the homologous to the E6-AP carboxyl terminus (HECT) family, Really Interesting New Gene (RING) family, and RING-in-between-RING (RBR) family (Morreale and Walden, 2016). Among these types, there are approximately 300 predicted RING E3s, making it the most abundant type of E3s (Li et al., 2008). The typical RING E3s contain a zinc-binding RING domain and function as monomers, homodimers, or heterodimers (Morreale and Walden, 2016). RING E3s typically function as a scaffold to recruit E2 in close proximity to substrate, thereby promoting direct transfer of Ub (Zheng and Shabek, 2017). Both monomeric and homodimeric U-box E3s belong to the RING type, despite the lack of zinc ions in its modified RING motif (Hatakeyama and Nakayama, 2003). The ubiquitination of HECT and RBR E3s involves a two-step reaction: (1) the transfer of Ub to the catalytic cysteine residue on E3s, and (2) the transfer of Ub from E3 to the target protein (Cotton and Lechtenberg, 2020; Wang et al., 2020b). Numerous E2s can function with a single E3 resulting in various outcomes, confirming that E2 significantly influences the outcomes of ubiquitination (Wenzel et al., 2011; Stewart et al., 2016). Over the last decade, research interest in DTX family E3s has increased, and considerable efforts have been made to study this family of RING type E3 ligases. The current available data suggest that the DTX family members are closely involved in cell growth, differentiation, apoptosis, intracellular signal transduction, as well as several diseases, including cancer. However, our knowledge of their substrates, biological and pathological functions, and exact molecular mechanisms is limited. In this review, we aim to provide a comprehensive view on characteristic structural features, functions and associated molecular mechanisms of DTX family proteins. Moreover, we highlight some perspectives for future investigations. The improved understanding of the impacts of DTX family proteins on development and disease may pave the way for their potential clinical applications as diagnostic and prognostic targets.

## Structural Features of DTX Family in Different Species

*Drosophila Melanogaster* is one of the most popular experimental animal models due to its relatively short life cycle, easy maintenance, and high homology to the human genome (Singh and Irvine, 2012). The *Drosophila* genome contains four sets of chromosomes, thus making it easy to use for genetic manipulation in research (Taormina et al., 2019). *Drosophila*’s sole *Deltex* gene is located on chromosome X and has four exons and three introns. The murine homologs (*MDTX* genes) contain four additional exons and introns, compared to *Drosophila Deltex* (Pampeno et al., 2001). In mammals, the encoded DTX family proteins comprise five members, namely DTX1, DTX2, DTX3, DTX3L, and DTX4 (Kishi et al., 2001; Takeyama et al., 2003; Chatrin et al., 2020). Compared with the amino acid sequences of *Drosophila* Deltex (Dx) protein, seven additional amino acids (amino acids 145–151) are found in MDTXs, and 82 additional amino acids occur in the N-terminal sequences of human DTXs (Pampeno et al., 2001). Furthermore, the vertebrate DTX proteins lack the polyglutamine sequences (amino acids 250-302 and 488-513) (Pampeno et al., 2001). The diverse amino acid sequences in different species may indicate some evolutionary characteristics of DTX family proteins. However, the biological relevance of these amino acid sequence variations of DTX family proteins are yet to be fully understood.

**FIGURE 1:**
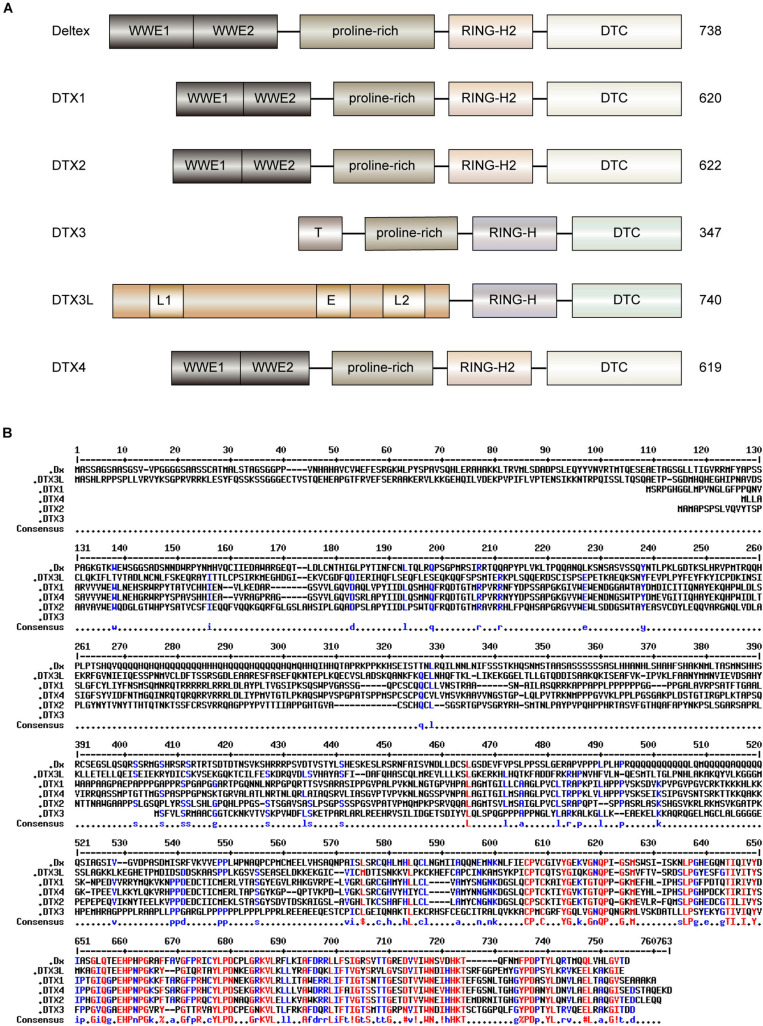
Domain architectures and sequence alignment of human DTX family and Dx protein in *Drosophila*. **(A)** The domain architecture of human DTX family and Dx. The functional motifs of human DTX family members are highly analogous to those of Dx in *Drosophila.* The N-terminal WWE1 and WWE2 domains of Dx, DTX1, DTX2, and DTX4 can bind to the ankyrin repeats of the Notch receptor. DTX3 has a truncated unique N-terminal domain (T) that lacks the ability to bind to ankyrin repeats. The long N-terminal region of DTX3L is associated with nuclear localization signals (L1 and L2) and nuclear export signal (E). The proline-rich regions are similar, except for DTX3L. The Deltex C-terminal (DTC) domains are practically conserved in all DTX proteins. The RING domain of each C-terminal region is divided into classical RING-H2 domain (Dx, DTX1, DTX2, and DTX4) or non-classical RING-H domain (DTX3 and DTX3L). **(B)** The sequence alignment of human DTX family and Dx. The multiple sequence alignment was performed using hierarchical clustering (Corpet, 1988), and generated via the Multalin program (version 5.4.1) with a high consensus value of 90% and a low consensus value of 50%. The identical residues are shown in red, and similar residues are in blue. Consensus symbol of!: anyone of IV, $: anyone of LM, %: anyone of FY, and #: anyone of NDQEBZ.

Deltex has three distinct domains (I, II, and III) from the N- to C-terminus. The N-terminal domain I of Dx comprises two WWE motifs, both of which bind to the ankyrin repeat sequences of Notch (Zweifel et al., 2005). The N-termini of human DTX1, DTX2, and DTX4 share homology with that of Dx. However, DTX3 cannot interact with Notch due to the truncated sequences in the N-terminus (Takeyama et al., 2003). Moreover, the N-terminus of DTX3L differs from the remaining DTX family members and contains both nuclear localization and export signals (Takeyama et al., 2003). Poly-adenosine diphosphate (ADP)-ribosylation (PARylation) is a PTM process by which ADP-ribose (ADPr) units, from nicotinamide adenine dinucleotide (NAD^+^), are added to targeted residues (Glu, Asp, Lys, Arg, or Ser) of a protein (Zhang et al., 2013; Martello et al., 2016). The WWE motifs of the DTX family proteins attach to complexes via recognizing *iso*-ADPr, the minimal subunit of PAR polymer, with a characteristic glycosidic bond (Aravind, 2001; He et al., 2012; Wang et al., 2012). Domain II of Dx contains a proline-rich motif, which is the binding site of the SH3 domain, that primarily regulates the interaction with other proteins, such as growth factor receptor-binding protein 2 (Grb2) (Matsuno et al., 1998). Lacking the proline-rich motif negatively reverses the Dx regulation of Notch signaling pathway (Matsuno et al., 2002). The C-terminal structures of *Drosophila* Dx, MDTXs, and human DTXs are highly evolutionarily conserved according to their amino acid sequence and crystal structure alignment (Kishi et al., 2001; Takeyama et al., 2003; Chatrin et al., 2020). The high sequence conservation across species suggests that DTX family proteins are likely to function in a similar manner, including binding to NAD^+^ (Chatrin et al., 2020). The C-terminus of Dx contains a RING-H2 domain with E3 ligase activity (Takeyama et al., 2003). In the integral steps of Dx regulated signaling pathway, the formation of homo-multimeric Dx is mediated by the RING-H2 domain (Matsuno et al., 2002). The RING-H2 domain of the DTX family adopts a novel circular fold with eight conserved cysteine and histidine residues, which is different from other RINGs (Miyamoto et al., 2019), whereas DTX3 and DTX3L contain a RING-HC structure with a single histidine (Takeyama et al., 2003). The Deltex C-terminal (DTC) domain, a relatively conserved novel fold and a close neighbor to the RING domain, has been reported in DTX family (Obiero et al., 2012). The functions of the DTC domain are poorly understood. The domain structures and sequence alignments of Dx and DTX family proteins are illustrated in Figure 1.

## Functions and Associated Molecular Mechanisms of the DTX Family

### Dx

**FIGURE 2:**
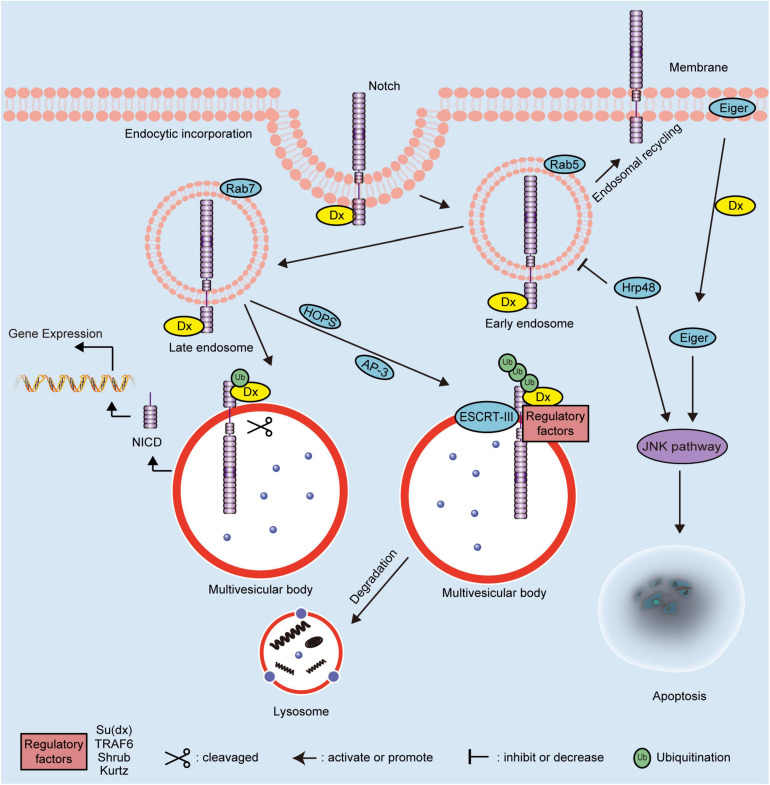
Schematic diagram showing the molecular mechanisms of Dx in *Drosophila*. The regulation mechanisms of Dx on the Notch signaling pathway depend on the ubiquitination pattern of the Notch receptor. Dx activates the endocytosis of Notch independent on the canonical ligands. The endosomal maturation is initiated by the HOPS complex and converted from Rab5 to Rab7. Interaction with the AP-3 complex promotes Notch targeting to the late-endosomal and lysosomal vesicle membranes. Dx functions as a positive modulator of the Notch signaling pathway when the Notch receptor is monoubiquitinated. The extracellular domain of monoubiquitinated Notch is removed and degraded following cleavage. Then, the NICD of Notch is released to activate the downstream gene expression. However, Dx acts as a negative regulator of the Notch signaling pathway when Notch is polyubiquitinated by Dx and regulatory factors, including Su(dx), TRAF6, Shrub, and Kurtz. The polyubiquitinated Notch is transferred into multivesicular body via ESCRT-III and degraded via the endosome/lysosome pathway. Dx also influences the JNK signaling pathway to induce apoptosis via interacting with Hrp48 or Eiger. NICD, intracellular domain; Dx, Deltex; TRAF6, tumor necrosis factor receptor associated factor 6; AP-3, adaptor protein-3; HOPS, homotypic fusion and vacuole protein sorting; ESCRT-III, endosomal sorting complex required for transport-III; Hrp48, heterogeneous nuclear ribonucleoprotein 48; JNK, Jun N-terminal Kinase; Su(dx), Suppressor of Deltex; Ub, ubiquitin.

The domain features of Dx influence its association with the Notch signaling pathway, one of the pivotal regulators of cell fate (Liang et al., 2019). The direct interactions between the cytoplasmic protein Dx and transmembrane receptor Notch have been previously demonstrated (Diederich et al., 1994). Upon Notch receptor activation, the intracellular domain of Notch receptors (NICD) is released, which translocates into the nucleus where it triggers the expression of the downstream genes (Kovall et al., 2017; Bray and Gomez-Lamarca, 2018). Dx interacts with the Notch receptor via the non-canonical signaling pathway in *Drosophila* (Hori et al., 2012). The established molecular mechanisms of Dx protein are illustrated in Figure 2. Dx overexpression induces morphological and phenotypic changes in *Drosophila*’s eyes, wings, and bristles, consistent with phenotypic changes induced by activation of NICD. Moreover, phenotypic changes caused by Dx inhibition could be partially rescued by an extra copy of Notch (Gorman and Girton, 1992). The classical Notch signaling pathway is activated prior to the Notch receptor entry into the multivesicular body, whereas Dx-mediated Notch signaling transduction is activated in a different manner (Yamada et al., 2011). It is established that endogenous Dx is necessary to: (1) assist Notch transport more efficiently from the plasma membrane into the endocytic vesicles, and (2) retain Notch on the surface of the late endosome, which prevents Notch trafficking to lysosomes for degradation (Yamada et al., 2011). Dx promotes the endocytosis and intracellular transport of Notch based on the activities of homotypic fusion and vacuole protein sorting (HOPS) and adaptor protein-3 (AP-3) complexes, which are regulated by Rab5 and Rab7 GTPases (Wilkin et al., 2008). Moreover, some evolutionarily conserved key transmembrane proteins, such as Crumbs, rely on Dx to modify the localization and trafficking of the Notch receptor (Nemetschke and Knust, 2016). In stellate cells, an expressional decrease or functional inhibition of Rab11 can lead to the accumulation of Notch receptors in early and late endosomes, thus activating Dx mediated non-canonical Notch signaling pathway (Choubey et al., 2020). During regulation of the endocytic trafficking of Notch, domains I and III of Dx are essential for stabilizing Notch in the late endosome (Hori et al., 2005).

Deltex has been shown to positively regulate the Notch signaling pathway (Xu and Artavanis-Tsakonas, 1990; Gorman and Girton, 1992). The interaction between Dx and Notch ankyrin repeats also interferes with the retention of the Suppressor of Hairless [Su(H)] in the cytoplasm and facilitates its translocation into the nucleus (Matsuno et al., 1995). In addition, Dx can solely promote monoubiquitination of the Notch receptor and triggers intracellular activation of Notch independent of canonical ligands (Hori et al., 2011). Neural precursor cell expressed developmentally down-regulated 4 (Nedd4), which contains a calcium/lipid-binding domain (C2 domain), two conserved tryptophan residues (WW domains), and a HECT domain, belongs to a family of HECT E3s (Kumar et al., 1992; Bork and Sudol, 1994; Boase and Kumar, 2015). The C2 domain in Nedd4 family is involved in protein-protein interactions and relocates target proteins to phospholipid membranes (Morrione et al., 1999; Plant et al., 2000; Dunn et al., 2004). The WW domains interact with phospho-serine/threonine residues of substrates (Sudol et al., 1995), while the HECT domain attaches activated Ub via an intermediate thioester bond, and catalyzes the attachment of Ub and a lysine on the substrate protein (Rotin and Kumar, 2009). Nedd4 suppresses the internalization and activation of Notch receptor by directly antagonizing Dx, further suggesting Dx as a positive modulator of the Notch signaling pathway (Sakata et al., 2004).

Interestingly, when interacting with additional proteins, such as Suppressor of deltex [Su(dx)] and Kurtz, Dx plays a negative regulatory role in the Notch signaling pathway. Su(dx), which belongs to the Nedd4 family E3, is a negative regulator of Notch (Mazaleyrat et al., 2003). Under normal circumstances, the WW domains and a linker region act synergistically to maintain Su(dx) in an autoinhibitory inactive state. Upon activation, Su(dx) induces the ubiquitination and degradation of Notch, while co-expression of Su(dx) and Dx blocks the activation of Notch signaling induced by Dx alone (Wilkin et al., 2008; Yao et al., 2018). Kurtz is the only homolog of non-visual beta-arrestin in *Drosophila* (Roman et al., 2000). Based on the results of yeast two-hybrid analysis, a region between amino acids 10 and 251 in Kurtz interacts with Dx, which leads to the polyubiquitination and degradation of Notch, thereby negatively regulating the Notch signaling pathway (Mukherjee et al., 2005). With the assistance of the core element, Shrub, of the endosomal sorting complex required for transport-III (ESCRT-III), the poly-ubiquitination of Notch is increased. Shrub and Dx shift the delivery of Notch receptor to multivesicular bodies, ultimately promoting the endosomal/lysosomal degradation of Notch (Hori et al., 2011). In addition, the proteins encoded by the *maheshvara* and *TNF receptor-associated factor 6* (*TRAF6*) are co-expressed with Dx to inhibit the Notch signaling pathway (Mishra et al., 2014; Surabhi et al., 2015). Therefore, the ubiquitination status (mono- or poly-ubiquitination) of Notch, mediated by Dx alone or in combination with any other possible interacting proteins, is correlated with the mechanisms underlying the effects of Dx on the downstream regulation pattern of Notch signaling pathway positively or negatively.

During homeostasis, cells integrate the activities of multiple pathways and turn on the interaction crosstalk, such as that between the Notch and c-Jun N-terminal kinase (JNK) signaling pathways (Ammeux et al., 2016). The synergistic interaction of heterogeneous nuclear ribonucleoprotein 48 (Hrp48) and Dx negatively regulates the Notch signaling pathway by inhibiting the transport of Notch from the cell membrane to the cytoplasm (Dutta et al., 2017). Additionally, The combinatorial expression of Hrp48 and Dx induces apoptotic cell death via the activation of the JNK signaling pathway (Dutta et al., 2019); similarly, Eda-like cell death trigger (Eiger) induces apoptosis by triggering JNK signal pathway (Igaki and Miura, 2014). Dx triggers the transport of Eiger from the cell membrane to cytoplasm and modulates its activity to induce the JNK signal pathway (Dutta et al., 2018). The cooperation of Dx and TRAF6 also mediates the Eiger-independent JNK activation, which is also regulated by the endocytic pathway component Rab7 (Sharma et al., 2021). Taken together, Dx has been shown to play a significant role in morphology and development of *Drosophila*, mainly by regulating JNK and non-canonical Notch signaling pathways. The understanding of the functions and molecular mechanisms of Dx in *Drosophila* establishes a quantitative framework for deeper research into mammalian DTX family proteins.

### MDTX Family of Proteins

Since conducting medical research on humans is restricted due to ethical issues and various other limiting factors, the laboratory mouse, *Mus musculus*, is one of the most commonly used mammals for studying human disease (Lloyd et al., 2016; Gurumurthy and Lloyd, 2019). The high genetic and physiological conservation are key advantages for mice as a suitable research animal model (Justice and Dhillon, 2016). The MDTX family of proteins have a high degree of similarity with Dx and their human homologs. In adult mice, the expression of MDTX1, MDTX3, and MDTX4 are prominently observed in the brain and testis, while MDTX2 is strongly expressed in the testis (Kishi et al., 2001; Storck et al., 2005). MDTX proteins inhibit the activity of a mammalian transcription factor E47 alone, rather than the E47-VP16 complex. In addition, overexpression of MDTX2 suppresses the expression of myogenic transcriptional factor *myogenin* and the frequency of muscle cell differentiation (Kishi et al., 2001). MDTX proteins can negatively regulate the Notch signaling pathway of T cells (Lehar and Bevan, 2006).

**FIGURE 3:**
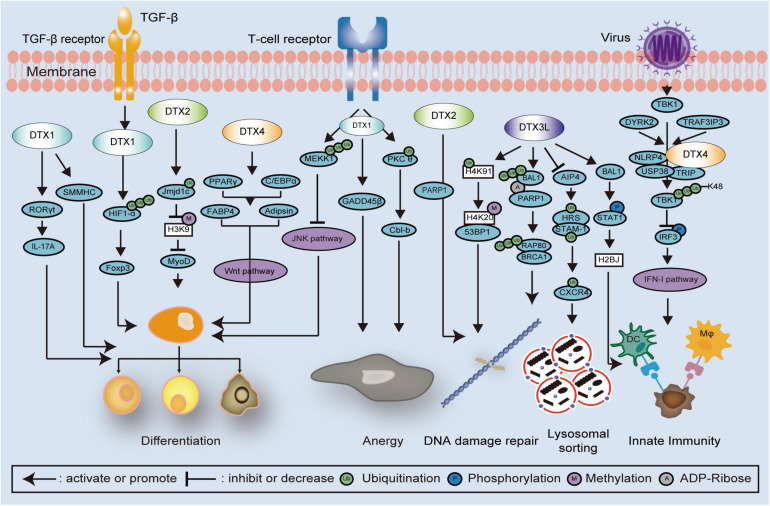
Schematic diagram showing DTX family related signaling pathways and interactions in cell development. DTX family proteins regulate cell differentiation via several mechanisms and signaling pathways, such as poly-ubiquitination of HIF1-α and MEKK1, monoubiquitination of Jmjd1c, methylation of histones, and JNK and Wnt signaling pathways. In the regulation of cell anergy, the T cell receptor is activated and monoubiquitination of PKCθ is induced by DTX1, following the alteration of GADD45 β and Cbl-b expression. In the regulation of DNA damage repair, DTX3L promotes the polyubiquitination of the RAP80-BRCA1 complex, monoubiquitination of histones, and STAT1phosphorylation. The combination of DTX2 and PARP1 is also involved with the regulation of DNA damage repair. Upon viral infection, TBK1 is phosphorylated thereby activating the IFN-I pathway. The DTX4, NLRP4, USP38, and TRIP complex inhibits the IFN-I pathway via enhancing polyubiquitination of TBK1, which is also associated with DYRK2 and TRAF3IP3. STAT1, signal transducer and activator of transcription 1; PARP1, Poly (ADP-Ribose) Polymerase 1; HIF1-α, hypoxia inducible factor 1 subunit alpha; MEKK1, mitogen activated protein kinase/ERK kinase 1; Jmjd1c, Jumonji domain containing 1c; JNK, Jun N-terminal Kinase; PKCθ, protein kinase Cθ; GADD45β, growth arrest and DNA damage inducible 45 beta; Cbl-b, Casitas B-lineage lymphoma-b; RAP80, receptor associated protein 80; BRCA1, Breast Cancer 1; TBK1, TANK binding kinase 1; NLRP4, nod-like receptor (NLR) family pyrin domain containing 4; USP38, Ub-specific protease 38; TRIP, TRAF-interacting protein; IFN-I, interferon type I; DYRK2, dual-specificity tyrosine-(Y)-phosphorylation-regulated kinase 2; TRAF3IP3, TNF receptor associated factor 3 interacting protein 3.

**FIGURE 4:**
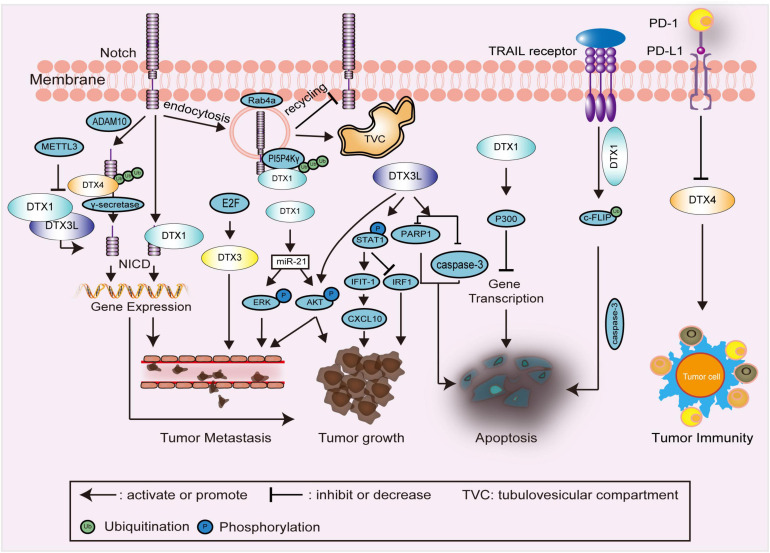
Schematic diagram showing DTX family related signaling pathways and interactions in carcinomas. In the regulation of Notch endocytosis, ubiquitination of PI5P4Kγ, as a substrate of DTX1, promotes the endocytosis and maintaining of Notch on the membrane of TVC, thereby restraining the recycling of Notch to the cell membrane. DTX family proteins, such as DTX1, DTX3L, and DTX4, are primarily involved in the regulation of the Notch signaling pathway and expression of downstream genes to affect tumor growth, and metastasis. In the regulation of tumor growth and metastasis, DTX1 and DTX3L also enhance the phosphorylation of downstream proteins, such as STAT1, AKT, and ERK. In the regulation of apoptosis, ubiquitination of c-FLIP by DTX1 stimulates TRAIL-induced cell death. The combination of P300 and DTX1 inhibits the expression of multiple genes, which is also associated with apoptosis. The expression of caspase-3 and PARP1 is decreased by DTX3L to inhibit apoptosis. In the regulation of tumor immunity, the expression of DTX4 is negatively regulated by PD-L1. NICD, intracellular domain of Notch receptor; PI5P4Kγ, phosphatidylinositol-5-phosphate 4-kinase γ; TVC, tubulovesicular compartment; STAT1, signal transducer and activator of transcription 1; c-FLIP, cellular FADD-like interleukin-1β converting enzyme inhibitory protein; TRAIL, TNF-related apoptosis-inducing ligand; PARP1, poly (ADP-Ribose) polymerase 1; PD-L1, programmed death ligand 1; METTL3, methyltransferase-like 3; ADAM10, a disintegrin and metalloproteinase 10; IFIT-1, interferon (IFN)-induced protein with tetratricopeptide repeats 1; IRF-1, IFN regulatory factor-1; CXCL10, Chemokine (C-X-C motif) ligand (CXCL)10.

In the sections that follow, the functions and associated molecular mechanisms of human DTX family proteins are summarized and reviewed. The related signaling pathways and interactions of DTX family proteins in cell development and in carcinomas are shown in Figures 3, 4, respectively. An overview of DTX family proteins during the developmental process of different cell types is listed in Table 1, while Table 2 summarizes the altered levels, functions, and mechanisms of DTX family proteins in different cancer types.

**TABLE 1:** Overview of studies with described functions and mechanisms of DTX family members during the development of different cell types.

Year	DTXs	Cell type	Results and findings
2002	DTX1	B cells	DTX1 antagonizes Notch1 signal pathway to induce the differentiation of lymphoid progenitor cells to B cells (Izon et al., 2002)
2003	DTX1	B cells	DTX1 is likely involved in the germinal center B cell differentiation (Gupta-Rossi et al., 2003)
2003	DTX1	B cells	DTX1 restrains Notch2 expression in the differentiation of marginal zone B cells (Saito et al., 2003)
1998	DTX1	T cells	Relative higher DTX1 expression and activated Notch signal pathway are detected in double negative and CD4^+^ and CD8^+^ single positive thymocytes, while both lower DTX1 expression and inactivated Notch signal pathway are detected in double positive thymocytes (Deftos et al., 1998)
2003	DTX1	T cells	DTX1 blocks hematopoietic stem cells to T lineage commitment, but not involved in early thymocyte development (Yun and Bevan, 2003)
2005	DTX1	T cells	DTX1 ubiquitinated MEKK1 and promotes its degradation to suppress the activation of T cells (Liu and Lai, 2005)
2006	DTX1	T cells	DTX1 competed with the binding of p300 to E2A/HEB protein, increasing survival of double positive thymocytes from the glucocorticoid-induced apoptosis (Jang et al., 2006)
2009	DTX1	T cells	DTX1 regulates the expression of anergy associated molecules, suppresses T cell activation, and participates in calcium-NFAT signal pathway to enhance T cell anergy (Hsiao et al., 2009)
2009	DTX1 DTX4	T cells	The combination of *DTX1* and *DTX4*, regulated by GATA3 in transcriptional level, interferes with Notch signal pathway during the early stage of T cell development (Wang et al., 2009)
2014	DTX1	T cells	DTX1 attenuates T cell activation and promotes the generation of T cell anergy by mono-ubiquitinating protein kinase C-θ, redirecting the localization of protein kinase C-θ and stabilizing Cbl-b (Hsu et al., 2014)
2015	DTX1	T cells	DTX1 degrades HIF-1α and enhances Foxp3 expression to maintain the stability of regulatory T cells (Hsiao et al., 2015)
2018	DTX4	T cells	The gene expression of *DTX4* is regulated by hsa_circ_0045272 to regulate apoptosis and interleukin-2 secretion of T cells in patients with systemic lupus erythematosus (Li et al., 2018)
2020	DTX1	T cells	DTX1 promoted the differentiation of CD4 ^+^ T cells into T helper 17 cells by enhancing the DNA binding ability of RORγt (Tang et al., 2020)
2020	DTX3L	Mononuclear cells	DTX3L advances phosphorylation of STAT1 and increases expression of CXCL10 to promote the infiltration of mononuclear cells (Tian et al., 2020)
2020	DTX4	Myeloid cells	DTX4 is regulated by TRAF3IP3 to decrease virus-triggered IFN-I production in myeloid cells (Deng et al., 2020)
2001	DTX1	Neural progenitor cells	DTX1 binds with transcription activator p300 and inhibits the activity of *MASH1* to restrain the differentiation of neural progenitor cells (Yamamoto et al., 2001)
2003	DTX1	Oligodendrocyte	F3/contactin initiates DTX1 dependent Notch signaling pathway to promote oligodendrocyte maturation and myelination (Hu et al., 2003)
2004	DTX1	Oligodendrocyte	NB-3, a member of the F3/contactin family, triggers Notch signal pathway via DTX1 to promote oligodendrocyte generation (Cui et al., 2004)
2005	DTX1	Bergmann glia	DNER mediated DTX1 dependent Notch signal to stimulate the morphological differentiation of Bergmann glial cells (Eiraku et al., 2005)
2018	DTX1	Muscle cells	DTX1 promotes the differentiation of smooth muscle cells by overexpressing the smooth muscle myosin heavy chain (MyHC) (Wang et al., 2018)
2018	DTX1	Muscle cells	DTX1 positively regulates the differentiation into smooth muscle cells to inhibit granulation tissue formation effectively for the treatment of closed penile fracture (Guo et al., 2018)
2017	DTX2	Muscle cells	DTX2 inhibits myogenic differentiation by suppressing the methylation of histone 3 of myogenic regulatory factor *MyoD* (Luo et al., 2017)
2020	DTX3L	Fibroblast like synoviocytes	DTX3L induces fibroblast like synoviocytes to produce inflammatory cytokines through STAT1 signal pathway (Hong et al., 2020)
2020	DTX1 DTX3L	Endothelial cells	The heterodimerization of DTX3L and DTX1 inhibits Notch signal pathway and ultimately restrains the angiogenesis of endothelial cells (Wang et al., 2020a)
2017	DTX4	Renal cells	The mRNA expression change of *DTX4* is regulated by microRNA let-7a and involved in the fibrotic processes of instructive nephropathy (Papadopoulos et al., 2017)
2017	DTX4	Preadipocytes	DTX4 upregulates the number of lipid granules, the expression of fat forming transcription factors, and adipogenic marker genes to increase differentiation of preadipocytes (Wang et al., 2017)
2017	DTX4	Hepatic cells	The DNA promoter methylation decrease of *DTX4* activates the differentiation of hepatic stellate cells (Schumacher et al., 2017)
2018	DTX4	Hepatic cells	DTX4 mediates IFN-I signal to influence HBV sustenance and maintenance of HBsAg in chronic hepatitis B (Kim et al., 2018)

**TABLE 2:** Overview of studies with described altered levels, functions, and mechanisms of DTX family members in different cancer types.

Year	DTXs	Cancer type	Expression	Results and findings
2012	DTX1	Splenic marginal zone lymphoma	Gene mutation	The mutation in WWE1 and proline-rich domains of *DTX1* occurs in splenic marginal zone lymphoma (Rossi et al., 2012)
2006	DTX3L	Diffuse large B-cell lymphoma	Up regulated	*DTX3L* is overexpressed in diffuse large B-cell lymphoma cells and shares the same bidirectional interferon-responsive promoter with *BAL1* (Juszczynski et al., 2006)
2013	DTX3L	Diffuse large B-cell lymphoma	Up regulated	DTX3L regulates the early Ub chain formation, RAP80 and BRCA1 recruitment to DNA damage sites in diffuse large B-cell lymphoma cells (Yan et al., 2013)
2014	DTX1	Diffuse large B-cell lymphoma	Gene mutation	*DTX1* mutations impair the inhibitory effects of Notch signal pathway in diffuse large B-cell lymphomas (de Miranda et al., 2014)
2017	DTX1	Diffuse large B-cell lymphoma	Gene mutation	*DTX1* with gene mutations plays tumor promoting roles in diffuse large B-cell lymphomas (Meriranta et al., 2017)
2017	DTX3L	Myeloma	Up regulated	DTX3L increases proliferation, adhesion, and chemo-resistance of myeloma cells, by blocking caspase-3 and PARP1 expression and inhibiting apoptosis (Shen et al., 2017)
2009	DTX1	Glioma	Up regulated	TSA increases the expression of DNER and DTX1 to abrogate growth and differentiation of glioblastoma derived neurospheres (Sun et al., 2009)
2013	DTX1	Glioma	Up regulated	DTX1 promotes the proliferation and invasiveness of glioblastoma cells and correlates with prognosis by activating the AKT and ERK pathways (Huber et al., 2013)
2017	DTX3L	Glioma	Up regulated	DTX3L is highly expressed in gliomas, relating to the malignant degree and the prognosis of patients (Xu et al., 2017)
2010	DTX1	Osteosarcoma	Down regulated	DTX1 inhibits invasiveness of osteosarcoma cells and negatively regulates Notch1 signaling (Zhang et al., 2010)
2018	DTX1	Gastric cancer	Down regulated	DTX1 decreases c-FLIP expression in lysosome dependent pathway and increases TRAIL-induced apoptosis in gastric cancer (Hsu et al., 2018)
2020	DTX3	esophageal carcinoma	Down regulated	DTX3 ubiquitinates Notch2 to suppress the proliferation and migration of esophageal carcinoma cells (Ding et al., 2020)
2011	DTX3 DTX4	Hepatocellular carcinoma	Up regulated	The E2F family transcription factors E2F1 and E2F3 binds directly to the proximal promoter regions of *DTX3* and *DTX4* to increase the levels of transcription in hepatocellular carcinoma cells (Viatour et al., 2011)
2010	DTX4	Colorectal cancer	Down regulated	*DTX4* is altered by a 1.6-fold change following treatment with Pomalidomide in colorectal cancer cells (Liu et al., 2010).
2019	DTX1	Non-small cell lung cancer	Gene mutation	The overall survival rate and disease-free survival rate of non-small cell lung cancer patients with *DTX1* gene mutation are both higher than those without *DTX1* mutation (Lee et al., 2019)
2020	DTX1	Small cell lung cancer	Gene mutation	The lower overall survival rate and worse response to chemotherapy are appeared in small cell lung cancer patients with *DTX1* gene mutation (Yoo et al., 2020)
2014	DTX3	Luminal subtype breast cancer	Up regulated	*DTX3* is essential for cell proliferation and uniquely amplified in highly proliferative luminal breast tumors (Gatza et al., 2014)
2020	DTX3	Triple-negative breast cancer	Down regulated	*DTX3* mRNA is degraded and its inhibitory effects on Notch4 is weaken, which promotes the metastasis of triple-negative breast cancer cells (Liu et al., 2020)
2020	DTX3L	Breast cancer	Up regulated	DTX3L is higher in breast cancers, especially in triple-negative breast cancer. DTX3L functions as a negative regulator of ATRA induced growth inhibition of breast cancer cells (Bolis et al., 2020)
2014	DTX3L	Prostate cancer	Up regulated	The overexpression of DTX3L enhances proliferation, metastasis, and chemo-resistance of prostate cancer cells by repressing the transcription of *IRF-1* and influencing phosphorylation of STAT1 (Bachmann et al., 2014)
2015	DTX3L	Melanoma	Up regulated	DTX3L regulates FAK/PI3K/AKT signal pathway to strengthen the invasion and metastasis of melanoma (Thang et al., 2015)
2016	DTX4	Melanoma	Up regulated	DTX4 is highly expressed as a Notch4 signaling pathway molecule in melanoma cancer stem like cells (Lin et al., 2016)
2016	DTX4	Nasopharyngeal carcinoma	Up regulated	The expression of DTX4 is higher in nasopharyngeal carcinoma cells (Liu et al., 2016)
2020	DTX4	Soft tissue sarcoma	Down regulated	The expression of *DTX4* in soft tissue sarcoma is regulated by IDO1 inhibitor combined with PD-L1 blockers (Nafia et al., 2020)

### DTX1

The human *DTX1* is located on chromosome 12 (12q24.13) and its 67.4 kDa coded protein contains 620 amino acids. DTX1 is 26% identical and 40% similar to Dx based on Needleman-Wunsch alignment of two protein sequences (Altschul et al., 1997). As for cellular location, it is located both in the cytoplasm and in the nucleus (Ordentlich et al., 1998; Yamamoto et al., 2001). The functions of DTX1 are determined by numerous factors. For example, during early development of thymocytes, a positive feedback loop has been reported between DTX1 upregulation and the activation of Notch signaling (Deftos et al., 1998). Meanwhile, a negative feedback between DTX1 and Notch is regulated by *HES1*, a downstream target gene of Notch, which directly binds to the promoter of *DTX1* and inhibits its transcription (Zhang et al., 2010). Atrophin-1-interacting protein 4 (AIP4) is another inhibitor of DTX1, which interacts with the proline-rich motif of DTX1 and mediates its degradation, primarily via K29-linked polyubiquitination and the lysosomal pathway (Chastagner et al., 2006). DTX1 was thought to directly bind to Notch and regulate its ubiquitination status, however, more recently, the regulation was found to be indirect (Zheng and Conner, 2018). The lipid kinase phosphatidylinositol-5-phosphate 4-kinase γ (PI5P4Kγ), as a substrate of DTX1, promotes Notch receptor internalization and localization in the tubulovesicular compartment via a Rab4a-dependent pathway, thus, preventing Notch receptor’s endosomal recycling back to the membrane and negatively regulating the Notch signaling pathway (Zheng and Conner, 2018).

DTX1 plays an essential role in cell differentiation. During avian development, DTX1 regulates the formation of the cranial neural crest via the Notch1 pathway (Endo et al., 2003). F3/contactin and its homolog NB-3 interact with Notch, thereby releasing the NICD via the non-canonical Notch pathway, and form a complex with DTX1 to mediate myelin-related protein expression in the nucleus (Hu et al., 2003; Cui et al., 2004). The neuron-specific transmembrane protein Delta/Notch-like epidermal growth factor-related receptor (DNER) mediates the interaction between neurons and glial cells via the DTX1 dependent Notch signaling pathway and promotes the morphological differentiation of Bergmann glial cells (Eiraku et al., 2005). DTX1, expressed in the nucleus of neural progenitor cells, directly interacts with the transcription activator p300, forming a complex that inhibits the transcriptional activity of *mammalian achaete–scute homolog 1 (MASH1)*, thereby restricting cell differentiation (Yamamoto et al., 2001). During differentiation of smooth muscle cells, DTX1 inhibits the proliferation of bone marrow mesenchymal stem cells and promotes their differentiation into smooth muscle cells by overexpressing smooth muscle myosin heavy chains (MyHCs) (Wang et al., 2018). DTX1 also effectively inhibits the formation of granulation tissue in the tunica albuginea, which is a treatment strategy used against closed penile fracture (Guo et al., 2018). During the development of lymphocytes, DTX1 induces lymphoid progenitor cells to differentiate into B cells, and is consistently involved in the differentiation of germinal-center B cells (Izon et al., 2002; Gupta-Rossi et al., 2003). During differentiation of marginal-zone B cells, DTX1 is overexpressed and restrains Notch2 expression (Saito et al., 2003). In addition, DTX1 inhibits the differentiation of hematopoietic stem cells into T cells (Yun and Bevan, 2003). T-lineage cells differentiate from multipotent progenitors, which exhibit different CD4 and CD8 phenotypes (Wu, 2006). During the early stages of T-cell development, the transcriptional level of *DTX1* is increased by the transcription factor GATA-binding factor 3 (GATA3); *DTX1* interferes with T-cell differentiation by regulating the Notch signaling pathway (Wang et al., 2009). The HeLa E box-binding (HEB) protein is often heterodimeric with E2A in thymocytes (Sawada and Littman, 1993). During maturation of CD4 and CD8 double-positive T cells, DTX1 competes with p300 for binding to the E2A/HEB protein complex, thereby enhancing the resistance of cells to glucocorticoid (GC)-induced apoptosis (Jang et al., 2006). DTX1 specifically promotes the degradation of the mitogen-activated protein kinase (MAPK)/ERK kinase 1 (MEKK1) via ubiquitination to inhibit T-cell activation (Liu and Lai, 2005). In addition to regulating the maturation of T cells, DTX1 also plays vital roles in the T-cell anergy process. DTX1 promotes the degradation of hypoxia-inducible factor-1α (HIF-1α) to maintain the expression of the transcription factor Forkhead box protein P3 (Foxp3), which is essential for sustaining the effector activities of regulatory T cells (Hsiao et al., 2015). Retinoic acid-related orphan receptor γ t (RORγt) is a transcription factor that is necessary for the differentiation of Th17 cells, CD4^+^ T helper lymphocytes that secrete interleukin (IL)-17A and IL-17F (Lee et al., 2020). When CD4^+^ T cells are stimulated by IL-6 and transforming growth factor-β (TGF-β), DTX1 promotes their differentiation into Th17 cells by enhancing the DNA-binding ability of RORγt in the nucleus and the production of the corresponding cytokines (Tang et al., 2020). The Casitas B-lineage lymphoma (Cbl) family is a RING type of E3 ligases, which acts as a negative regulator of immune activation (Liu and Gu, 2002). The mammalian Cbl family contains three homologs, namely c-Cbl, Cbl-b, and Cbl-c (Jafari et al., 2021). During the T-cell anergy process, DTX1 acts as a Notch-independent regulator, which induces the degradation of protein kinase C-θ (PKC-θ) by promoting its mono-ubiquitination and the endosome/lysosome pathway. Thus, the protein stability of Cbl-b increases to attenuate T-cell activation and promote anergy (Hsu et al., 2014). Upon induction by nuclear factor of activated T cells (NFAT), DTX1 regulates the expression of other anergy-associated molecules such as growth arrest and DNA-damage-inducible 45 beta (GADD45β) during the T-cell anergy process (Hsiao et al., 2009).

DTX1 also plays a pivotal role in tumorigenesis, invasion, and metastasis of several cancers. Overexpression of DTX1 increases the clonal ability, growth potential, and invasiveness of glioblastoma cells. Patients with low expression of DTX1 have a longer survival and a better prognosis of glioblastoma. DTX1 triggers a specific transcription process, including microRNA-21 and antiapoptotic *Mcl-1*, which are involved in the activation of the AKT and ERK pathways (Huber et al., 2013). In addition, induction of DNER by the histone deacetylase inhibitor trichostatin A (TSA) has been shown to reduce the tumorigenicity and cell differentiation of glioblastoma-derived neurosphere lines via the DTX1-mediated non-canonical Notch signaling pathway (Sun et al., 2009). Furthermore, DTX1 plays a tumor-suppressive role and is negatively associated with gastric cancer progression. In gastric cancer cells, DTX1 primarily promotes the degradation of cellular FADD-like IL-1β-converting enzyme-inhibitory protein (c-FLIP) in the lysosomal pathway and enhances TNF-related apoptosis-inducing ligand (TRAIL)-induced cell death (Hsu et al., 2018). Missense or nonsilent *DTX1* mutations have been reported in splenic marginal zone lymphomas and in Chinese patients with primary and recurrent diffuse large B-cell lymphomas (DLBCLs) (Rossi et al., 2012; de Miranda et al., 2014; Green et al., 2015). Almost all these mutations occur in the WWE domains of *DTX1* and impair its function as a negative Notch regulator, thereby promoting the development of DLBCLs (Meriranta et al., 2017). Mutations in the promoter region of *DTX1* were detected during early non-small-cell lung cancer (NSCLC); both overall survival (OS) and disease-free survival (DFS) rates were higher in patients with mutations than in those without mutations, suggesting that *DTX1* mutations were beneficial for the survival and prognosis of patients with early NSCLC (Lee et al., 2019). In contrast, patients with small cell lung cancer (SCLC) carrying *DTX1* mutations showed a worse response to chemotherapy and a lower OS rate, suggesting that mutations in the same gene may play opposite roles in different subtypes of malignant tumors in the same organ. The specific mechanisms underlying these mutations remain to be determined (Yoo et al., 2020).

### DTX2

Human *DTX2*, located on chromosome 7 (7q11.23), encodes a 67.2 kDa intranuclear protein with 622 amino acids. DTX2 is 26% identical and 38% similar to Dx following the comparison of the two protein sequences. PAR polymerase 1 (PARP1), activated by DNA damage, promotes PAR chain formation on many target proteins, including DTX2 (Jungmichel et al., 2013; Gupte et al., 2017; Ray Chaudhuri and Nussenzweig, 2017). The catalytic DTX2 is then recruited to promote ubiquitination of its targets at DNA damage sites. It was recently reported that the DTC domain of DTX2, not the WWE domains, played an essential role in binding PARylated substrate proteins and facilitated ubiquitination of substrate proteins by the RING domain (Ahmed et al., 2020). As the sequences of DTC domains are very similar in DTX family of proteins, almost all members, theoretically, can attach to PARylated proteins. Data from proteomics show that each DTX family protein has a specific protein interaction network (Ahmed et al., 2020). The results also suggest that 2,087 peptides, corresponding to 1,035 proteins, could be ubiquitinated by DTX2. In addition, only DTX2 showed a strong correlation with 71 DNA damage repair proteins. This diversity is partly attributed to the different cellular localization of DTX family proteins (Ahmed et al., 2020). The effects of DTX2 on cell differentiation have been demonstrated. Upon *DTX2* knock-out, skeletal muscle stem cells undergo early myogenic differentiation and accelerated regeneration in response to injury. In this process, DTX2 changes the methylation status of H3K9 in the distal regulatory region of the *MyoD* promoter and directly inhibits demethylase activity of Jumonji domain-containing 1C (JMJD1C) by monoubiquitination to reduce *MyoD* expression (Luo et al., 2017).

### DTX3

Human *DTX3* is located on chromosome 12 (12q13.3). The 38.0 kDa DTX3 protein has 347 amino acids and is primarily expressed in the nucleus. DTX3 is only 16% identical and 24% similar to Dx. The roles of DTX3 in tumor development have been extensively investigated. For example, a knockout of three retinoblastoma family genes in the liver of adult mice induced the development of liver tumors, similar to human hepatocellular carcinoma. In this model, the overexpression of *DTX3* was activated by the E2F family transcription factors E2F1 and E2F3 (Viatour et al., 2011). In ductal breast cancer, the amplification of *DTX3* is correlated with high proliferation of tumor cells and a poor prognosis (Gatza et al., 2014). Meanwhile, the expression of DTX3 in esophageal cancer tissue and cell lines is abnormally downregulated. DTX3 inhibits the proliferation and tumorigenicity of esophageal cancer cells and promotes the ubiquitination and degradation of Notch2 (Ding et al., 2020). Furthermore, *DTX3* acts as a tumor suppressor gene in triple-negative breast cancer and is expressed at a low level, which hinders its ubiquitination and degradation ability toward Notch4 and its ability to effectively inhibit triple-negative breast cancer metastasis (Liu et al., 2020).

### DTX3L

Human *DTX3L*, also known as B-lymphoma and B-aggressive lymphoma (BAL)-associated protein (BBAP), is located on chromosome 3 (3q21.1). The 83.6 kDa DTX3L protein has 740 amino acids. Protein sequence comparison results showed that DTX3L is 21% identical and 36% similar to Dx. *MDTX3L* is highly expressed in multiple organs and tissues, such as the thymus, hypothalamus, anterior pituitary gland, olfactory bulb, nose, mouth, urogenital sinus, and rectum (Hakme et al., 2008). DTX3L was originally identified as a binding partner of BAL1 (PARP9/ARTD9), which is an oncogenic factor in DLBCL with a prominent immune/inflammatory infiltrate (Juszczynski et al., 2006). Both *DTX3L* and *BAL1* are located on chromosome 3q21 in a head-to-head orientation and share the same bidirectional interferon (IFN)-responsive promoter (Juszczynski et al., 2006). The PARylation of protein is abundant at DNA lesion sites and critical for participating in the DNA damage repair pathways (Wei and Yu, 2016; Liu et al., 2017). PARP9 alone, without enzymatic activity, is unable to enzymatically active the PARylation of target proteins (Vyas et al., 2014). The presence of the DTX3L/PARP9 heterodimer, shuttling between the nucleus and cytoplasm and targeting proteins within the nucleosome, brings about the possibility that their functions are coupled in some way (Juszczynski et al., 2006). The heterodimer of DTX3L/PARP9 displays the PARylation activity, which requires E1, E2, and ATP, by cleaving NAD^+^ and generating ADPr. Ub is observed to be mono-ADP-ribosylated with the ADPr, which produced from DTX3L/PARP9 reaction. The ADP-ribosylated modification of Ub occurs on C-terminal Gly^76^, which is an important residue for the formation of polyUb chain. As a result, ADP-ribosylation of Ub strongly reduce polyUb formation while has no obvious effect on monoubiquitination of target proteins (Yang et al., 2017). Recently, it is unexpectedly found that the ADP-ribosylation of Ub happens independent of PARP9. DTX3L alone can transfer ADPr directly to Ub. The DTC and RING domains, when together, are the minimum fragments required of the DTX family proteins for catalyzing ADP-ribosylation of Ub. In ADP-ribosylation of Ub, the DTC domain accommodates NAD^+^ while the RING domain is responsible for recruiting E2∼Ub; conformational arrangement of these two domains is essential (Chatrin et al., 2020). DTX3L catalyzes the monoubiquitination of histone H4K91 and promotes the binding of methylated histone H4K20 to 53BP1 during DNA damage response (Yan et al., 2009). Breast Cancer 1 (BRCA1) protein is a RING type of E3 ligase, consisting of C-terminal BRCT motifs and a N-terminal RING domain, and plays a key role during checkpoint modulation and DNA damage repair (Scully and Livingston, 2000; Xu et al., 2001; Yarden et al., 2002; Zhang et al., 2004; Zhuang et al., 2006). BRCA1 interacts with different adaptor proteins, including receptor-associated protein 80 (RAP80), and forms complexes with distinct functions for DNA repair (Kim et al., 2007; Sobhian et al., 2007; Yan et al., 2007). The early Ub chain formation and the recruitment of RAP80 and BRCA1 to DNA damage sites are dependent on the colocalization of PARP1, BAL1, and DTX3L (Yan et al., 2013). In addition, DTX3L directly interacts with AIP4 and limits the ubiquitination of ESCRT-0 subunits, hepatocyte growth factor receptor tyrosine kinase substrate (HRS), and signal transducing adaptor molecule (STAM), which regulate the maintenance of ESCRT-0 on early endosomes to sort ubiquitinated chemokine (C–X–C motif) receptor 4 (CXCR4) for lysosomal degradation (Holleman and Marchese, 2014).

Several studies have shown that the expression of DTX3L is associated with inflammatory diseases. For instance, during viral infection, the PARP9/DTX3L complex targets histone H2BJ by interacting with signal transducer and activator of transcription 1 (STAT1) (Zhang et al., 2015). Meanwhile, in rheumatoid arthritis, DTX3L induces fibroblast-like synoviocytes (FLS) to produce inflammatory cytokines via the STAT1 signal transduction pathway (Hong et al., 2020). A low level of RNA N^6^-methyladenosine methyltransferase methyltransferase-like 3 (METTL3) has been reported in tissues of cerebral arteriovenous malformations. METTL3 modulates the mRNA stability of DTX3L and inhibits the heterodimerization of DTX3L and DTX1. It consequently promotes the downstream gene expression of the Notch signaling pathway and ultimately accelerates the angiogenesis of endothelial cells (Wang et al., 2020a). In primary Sjogren’s syndrome, the DTX3L/BAL1 complex enhances the phosphorylation of STAT1 to upregulate IFN-induced protein with tetratricopeptide repeats 1 (IFIT-1) and increases the expression of chemokine (C–X–C motif) ligand 10 (CXCL10), thereby promoting the infiltration of mononuclear cells (Tian et al., 2020).

The overexpression of DTX3L in multiple carcinomas has been previously investigated. In lymphoma, the high expression level of DTX3L contributes to the resistance to DNA-damaging chemotherapeutic agents (Yan et al., 2013). The overexpression of DTX3L and BAL1 promotes the phosphorylation of STAT1 and represses the transcription of *IFN regulatory factor-1 (IRF-1)*, thus enhancing the proliferation, metastasis, and chemoresistance of prostate cancer cells (Bachmann et al., 2014). DTX3L is also highly expressed in gliomas, and its expression level correlates with the degree of malignancy and the overall prognosis (Xu et al., 2017). The regulatory mechanism underlying the invasion and metastasis of melanoma by DTX3L involves the focal adhesion kinase (FAK)/PI3K/AKT signal transduction, but not the MEK/ERK pathway (Thang et al., 2015). The expression of DTX3L is regulated by FAK and gradually increases during proliferation of myeloma cells, which results in cell cycle arrest at the G1 phase and promotes the adhesion of myeloma cells to fibronectin or bone marrow stromal cells (Shen et al., 2017). Meanwhile, inhibition of DTX3L expression has been shown to enhance the sensitivity to chemotherapy and increase the expression of caspase-3 and PARP1 in multiple myeloma cell lines, thus promoting apoptosis (Shen et al., 2017). Furthermore, DTX3L expression is higher in triple-negative breast cancer cells than in estrogen receptor (ER) positive and human epidermal growth factor receptor 2 (HER2) positive breast cancers, and is a part of the negative feedback loop controlling all-trans retinoic acid (ATRA)-dependent inhibition of breast cancer cell growth (Bolis et al., 2020).

### DTX4

*DTX4*, as the last discovered member of the DTX family, is located on chromosome 11 (11q12.1). The 67.4 kDa DTX4 protein with 619 amino acids is primarily expressed in the cytoplasm, and is 27% identical and 39% similar to Dx. DTX4 is closely involved in the Notch signaling pathway. After Notch1 is ubiquitinated by DTX4 on the cell surface, ligand-expressing cells internalize the extracellular domain of Notch1. At the same time, Notch1 receptor-expressing cells internalize the complex of Notch1 and DTX4 in a process referred to as bilateral endocytosis (Chastagner et al., 2017). A disintegrin and metalloproteinase 10 (ADAM10) generates a cleavage product of Notch, necessary for the NICD formation. Blocking endocytosis of Notch1 and DTX4 reduces the colocalization of Notch1 and ADAM10 and the formation of the NICD, which suggests that DTX4 ubiquitinates Notch1 prior to the cleavage by ADAM10 (Chastagner et al., 2017).

In addition to Notch signaling, DTX4 is also involved in IFN-I signaling pathway in innate immunity. In virus-infected cells, IRF-3 is phosphorylated by TANK-binding kinase 1 (TBK1), thereby activating the IFN-I signaling pathway. The Ub-specific protease 38 (USP38), TRAF-interacting protein (TRIP), Nod-like receptor (NLR) family pyrin domain containing 4 (NLRP4), and DTX4 complex polyubiquitinates TBK1, thereby degrading it to limit the virus-induced IFN-I signaling pathway (Cui et al., 2012). Some interacting proteins, such as TNF receptor-associated factor 3-interacting protein 3 (TRAF3IP3) and dual-specificity tyrosine-(Y)-phosphorylation regulated kinase 2 (DYRK2), are also essential for the NLRP4/DTX4 complex to promote TBK1 degradation via Lys48-linked ubiquitination (An et al., 2015; Deng et al., 2020). In chronic hepatitis B, the reduction of DTX4 expression partially mediates the IFN-I signaling pathway to increase the sustenance of hepatitis B virus (HBV) and maintenance of hepatitis B surface antigen (HBsAg) in the serum (Kim et al., 2018).

DNA promoter methylation negatively correlates with gene expression. With the decrease in its DNA promoter methylation, *DTX4* expression is promoted during the activation of hepatic stellate cells (Schumacher et al., 2017). In systemic lupus erythematosus, the mRNA expression of *DTX4* is partially modulated by circular RNA hsa_circ_0045272 and is associated with early apoptosis of Jurkat cells (Li et al., 2018). *DTX4* is also involved in fibrotic processes in obstructive nephropathy, and its mRNA levels are regulated by microRNA let-7a (Papadopoulos et al., 2017).

DTX4 plays vital roles in cell differentiation. The elevated expression of *DTX4*, together with *DTX1*, has been shown to contribute to their inhibitory effects on Notch signaling pathway. As a result, T-cell commitment and developmental progression are impeded (Wang et al., 2009). During preadipocyte differentiation, the expression of DTX4 protein gradually increases. Then, the artificially reduced expression of DTX4 is found to decrease the number of lipid granules, along with the decreased expression of CCAAT enhancer-binding protein alpha (C/EBPα) and peroxisome proliferator-activated receptor gamma (PPARγ). Moreover, downregulation of DTX4 reduces the expression of adipogenic marker genes *fatty acid-binding protein 4 (FABP4)* and *adipsin*, which arrest mitosis and inhibit expression of Wnt signaling genes, such as *Wnt6* and *Wnt10b* (Wang et al., 2017).

DTX4 is associated with the development and metastasis of several carcinomas, such as hepatocellular carcinoma (Viatour et al., 2011), colorectal cancer (Liu et al., 2010), and melanoma (Lin et al., 2016). Comparison of the interaction networks between microRNAs and target genes in nasopharyngeal carcinoma samples showed that *DTX4*, regulated by several microRNAs, was substantially upregulated, which illustrates the promotional roles of *DTX4* in the occurrence and development of nasopharyngeal carcinoma (Liu et al., 2016). Indoleamine 2,3 dioxygenase (IDO1), the rate-limiting enzyme of the kynurenine pathway, and programmed cell death 1 ligand 1 (PD-L1) are potential immunotherapeutic targets against soft tissue sarcoma. The expression of *DTX4* increases upon the inhibition of both IDO1 and PD-L1, which suggests the potential controlling function of *DTX4* in immunotherapy of heterogeneous malignant mesenchymal neoplasms (Nafia et al., 2020).

## Conclusion

DTX family E3 ligases are highly evolutionarily conserved and essential during protein ubiquitination, yet differ from each other with various functions in the expressed tissues. DTX1, expressed in both cytoplasm and nucleus, has the highest homology with Dx of *Drosophila*. The structure, function, and mechanism of DTX1 remains a hot topic in research. DTX1 activates multiple signaling cascades to regulate cell development, while dysregulated DTX1 expression induces numerous human diseases, including malignant conditions. Several substrate proteins of DTX1 have already been identified, for example, PI5P4Kγ, c-FLIP, and PKC-θ. The cellular localizations of DTX2, DTX3, and DTX3L are primarily in the nucleus, indicating that the functions and mechanisms of these three DTX proteins are associated with transcriptional regulation and DNA damage repair. The mechanism of DTX2 involves PARylation. The N-termini of DTX3 and DTX3L are disparate from those of other DTX proteins, while the current available data on DTX3 are limited. DTX3L and PARP9 heterodimer targets proteins within the nucleosome. DTX4 is the last discovered member and primarily expressed in the cytoplasm and is involved in human innate immune by regulating IFN-I signaling pathway. Owing to the complexity of multiple E2 and substrate proteins, the function and mechanism of DTX family proteins remain nebulous. Further research can provide deeper insights into ubiquitination. Currant data suggest DTX proteins as potential diagnostic and therapeutic targets for carcinomas and other diseases.

## Future Perspectives

It is well accepted that the protein structure determines function. In the future, with the help of AlphaFold, an artificial intelligent system to predict the 3-D structure of a protein accurately, the structural features, molecular mechanisms, and potential drug targets of DTX family proteins will be no longer mysterious (Jumper et al., 2021). The DTX family proteins have a great significance in both physiology and pathology, hence further research is warranted to elucidate the mechanisms underlaying their function and influence, such as: (1) What other substrate proteins are directly ubiquitinated by DTX family proteins? The fundamental function of DTX family proteins is the ubiquitination of substrate proteins. Although some have been identified, many substrate proteins of DTX family have yet to be fully characterized. Binding specificity of substrate proteins, to a certain extent, determines the exact molecular mechanism and downstream signaling pathway. Further investigation will provide a better understanding of functional roles of individual DTX proteins. (2) What results in the aberrant expression of DTX family proteins in carcinoma and other diseases? Genetic mutation and transcriptional dysregulation are associated with the over- or down- expression of DTX family proteins under pathological conditions. However, the exact mechanisms remain to be determined. (3) What is the relationship between other E3s and DTX family proteins? Several other E3s, for instance, AIP4, Nedd4, and BRCA1, play different roles in enhancement or inhibition of the ubiquitination by DTX family proteins. Both extracellular stimuli and intracellular conditions influence the combination of other E3s with DTX proteins, which are extremely complicated and require in depth investigation. It is of great importance to identify the association between other E3s and DTX proteins, which will provide insights into translational medicine of DTX proteins.

## Author Contributions

LW and ZL concepted and designed the review. LW wrote the manuscript. LW, XS, and JH revised the manuscript. All authors contributed to the article and approved the submitted version.

## Conflict of Interest

The authors declare that the research was conducted in the absence of any commercial or financial relationships that could be construed as a potential conflict of interest.

## Publisher’s Note

All claims expressed in this article are solely those of the authors and do not necessarily represent those of their affiliated organizations, or those of the publisher, the editors and the reviewers. Any product that may be evaluated in this article, or claim that may be made by its manufacturer, is not guaranteed or endorsed by the publisher.
